# Development of a combined radiomics and CT feature-based model for differentiating malignant from benign subcentimeter solid pulmonary nodules

**DOI:** 10.1186/s41747-023-00400-6

**Published:** 2024-01-17

**Authors:** Jianing Liu, Linlin Qi, Yawen Wang, Fenglan Li, Jiaqi Chen, Shulei Cui, Sainan Cheng, Zhen Zhou, Lin Li, Jianwei Wang

**Affiliations:** 1 Department of Diagnostic Radiology, National Cancer Center/National Clinical Research Center for Cancer/Cancer Hospital, Chinese Academy of Medical Sciences and Peking Union Medical College, No. 17 Panjiayuan Nanli, Chaoyang District, Beijing, 100021 China; 2 Beijing Deepwise & League of PhD Technology Co. Ltd, Beijing, China

**Keywords:** Diagnosis (differential), Machine learning, Nomograms, Solitary pulmonary nodule, Tomography (x-ray computed)

## Abstract

**Background:**

We aimed to develop a combined model based on radiomics and computed tomography (CT) imaging features for use in differential diagnosis of benign and malignant subcentimeter (≤ 10 mm) solid pulmonary nodules (SSPNs).

**Methods:**

A total of 324 patients with SSPNs were analyzed retrospectively between May 2016 and June 2022. Malignant nodules (*n* = 158) were confirmed by pathology, and benign nodules (*n* = 166) were confirmed by follow-up or pathology. SSPNs were divided into training (*n* = 226) and testing (*n* = 98) cohorts. A total of 2107 radiomics features were extracted from contrast-enhanced CT. The clinical and CT characteristics retained after univariate and multivariable logistic regression analyses were used to develop the clinical model. The combined model was established by associating radiomics features with CT imaging features using logistic regression. The performance of each model was evaluated using the area under the receiver-operating characteristic curve (AUC).

**Results:**

Six CT imaging features were independent predictors of SSPNs, and four radiomics features were selected after a dimensionality reduction. The combined model constructed by the logistic regression method had the best performance in differentiating malignant from benign SSPNs, with an AUC of 0.942 (95% confidence interval 0.918–0.966) in the training group and an AUC of 0.930 (0.902–0.957) in the testing group. The decision curve analysis showed that the combined model had clinical application value.

**Conclusions:**

The combined model incorporating radiomics and CT imaging features had excellent discriminative ability and can potentially aid radiologists in diagnosing malignant from benign SSPNs.

**Relevance statement:**

The model combined radiomics features and clinical features achieved good efficiency in predicting malignant from benign SSPNs, having the potential to assist in early diagnosis of lung cancer and improving follow-up strategies in clinical work.

**Key points:**

• We developed a pulmonary nodule diagnostic model including radiomics and CT features.

• The model yielded the best performance in differentiating malignant from benign nodules.

• The combined model had clinical application value and excellent discriminative ability.

• The model can assist radiologists in diagnosing malignant from benign pulmonary nodules.

**Graphical Abstract:**

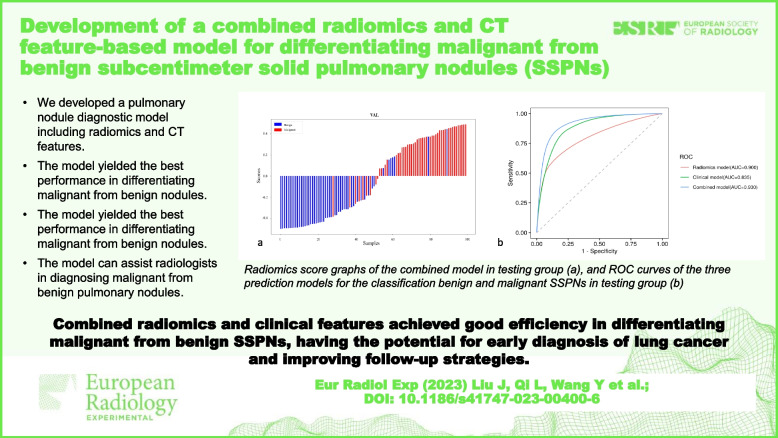

## Background

Lung cancer is the leading cause of cancer-related death in the world, accounting for 18.0% of the total cancer deaths [1]. According to the eighth edition of the tumor, node, and metastasis classification for lung cancer, the 5-year survival rate of patients with stage IA1 lung cancer is 90%, while it drops to 12% in stage IIIC [2], which reveals that early screening and diagnosis of lung cancer are essential. Early-stage lung cancer usually presents as solitary pulmonary nodules, which can be divided into solid nodules and sub-solid nodules based on density [3]. Malignant solid nodules show higher grade malignancy, earlier metastasis, and worse prognosis [4–7]. In addition, malignant solid nodules have a short doubling time and rapid growth [8].

Under T staging, subcentimeter nodules are the smallest nodules. If malignant solid nodule can be diagnosed and treated in time at subcentimeter stage, it will effectively improve the prognosis of patients. Besides, despite the small size of the tumor, solid subcentimeter non-small cell lung cancer does not always correspond to early-stage disease [9]. Malignant subcentimeter solid pulmonary nodules (SSPNs) can present lymph node metastasis and distant metastasis, and lymphatic, vascular, and pleural invasion are also more likely to occur in patients with solid subcentimeter NSCLC [9, 10]. However, the differential diagnosis of SSPNs is particularly difficult in clinical practice. There is an overlap of benign and malignant SSPNs in computed tomography (CT) imaging as some small nodules lack obvious imaging characteristics [11]. Biopsy is very difficult for SSPNs and prone to false negatives, and follow-up may cause additional radiation exposure and psychological and financial burden for patients [12, 13]. More importantly, the delayed diagnosis of malignant solid nodules may lead a patient’s prognosis to further deteriorate. Therefore, a more accurate and earlier diagnostic approach is essential for patients with SSPNs.

Radiomics can extract a large number of high-dimensional imaging features and convert this imaging information into quantitative parameters for analysis and modeling [14], which may serve as a noninvasive method to support personalized clinical decision-making. Previous studies have revealed that radiomics has great potential to support radiologists in identifying benign and malignant solid pulmonary nodules [11, 15–18], but few studies have investigated the performance of enhanced CT radiomics in differentiating malignant from benign SSPNs. We aimed to explore the value of enhanced CT-based radiomics in discriminating malignant from benign SSPNs, to develop a combined model based on clinical and radiomics features for the differential diagnosis of SSPNs in the clinic.

## Methods

### Patient selection

**Fig. 1:**
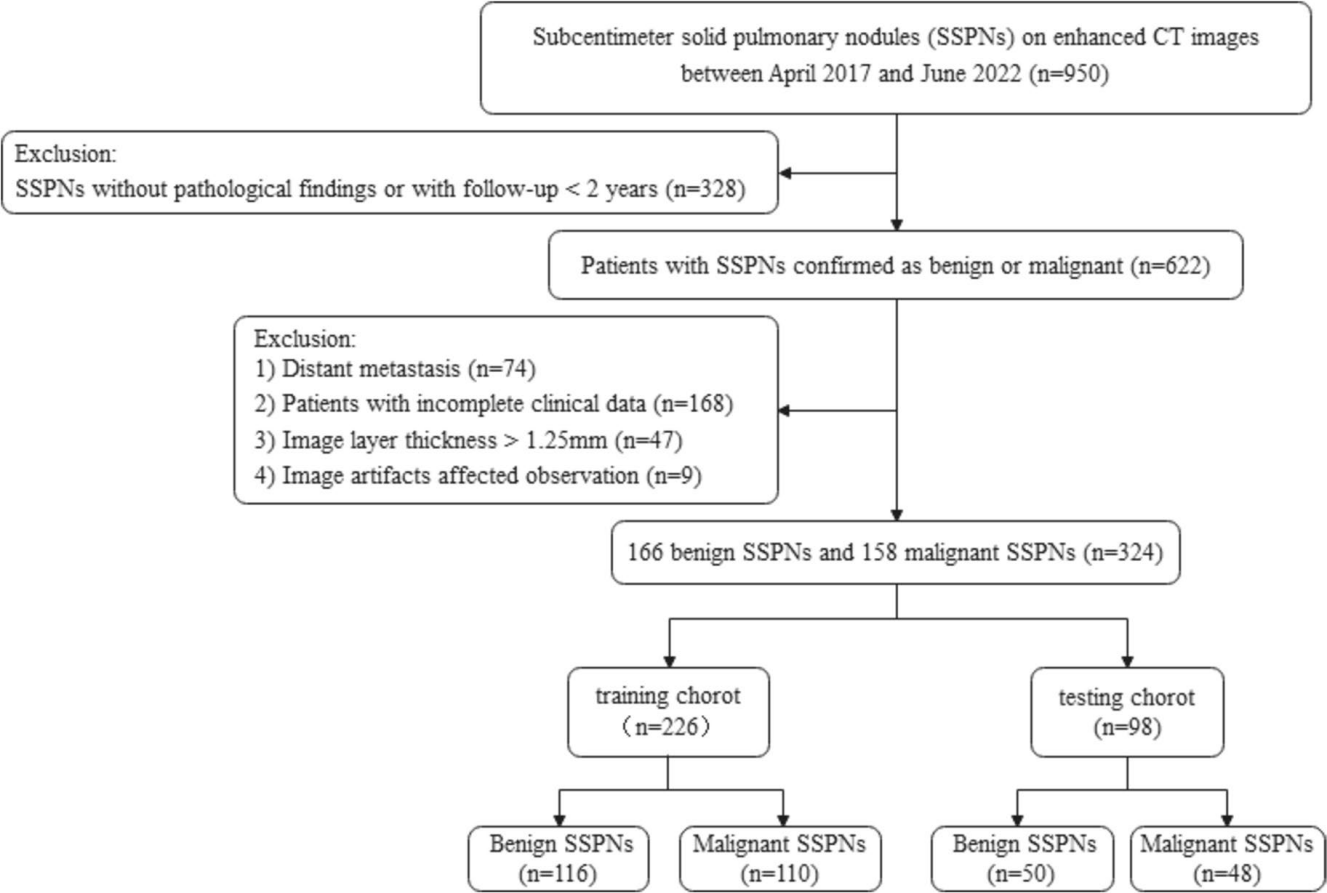
The flow chart showing the patient recruitment process. *SSPNs*, Subcentimeter solid pulmonary nodules

The institutional review board waived the requirement for informed patient consent for this retrospective study. We retrospectively reviewed 950 patients with SSPNs on enhanced CT in our hospital from April 2017 to June 2022. A total of 324 patients were included, based on the following criteria: (1) mean solid nodule diameter ≤ 10 mm; (2) malignant and benign nodules confirmed by surgical pathology, with nodules that remained stable for more than 2 years after follow-up or that became smaller or disappeared for less than 2 years of follow-up considered benign; and (3) enhanced CT images with slice thickness ≤ 1.25 mm. The exclusion criteria were as follows: (1) metastases confirmed histologically by surgical resection, (2) poor image quality or evident artifacts on CT images, and (3) patients with incomplete clinical data. The 324 patients with 324 nodules were randomly divided into the training (*n* = 226) and testing (*n* = 98) cohorts, according to a 7:3 ratio, for model learning. The study workflow is shown in Fig. 1.

### CT protocol

Chest CT images were obtained using 64-detector (LightSpeed VCT or Optima CT660, General Electric Medical Systems, Milwaukee, WI, USA; Toshiba Aquilion, TOSHIBA Medical Systems, Otawara, Japan) multislice scanners and were reconstructed using standard algorithms. The parameters of LightSpeed VCT or Optima CT660 were as follows: tube voltage, 120 kVp; auto mA settings (tube current 120–500 mA; noise index 11 or 13; helical pitch 0.992 or 0.984; rotation time 0.5 or 0.6 s; thickness 5 mm), and the parameters of Toshiba Aquilion were reconstructed at 120 kV and 150 mAs, with a helical pitch of 0.980 s and a thickness of 5 mm. Reconstruction thicknesses were 1.00 or 1.25 mm at a 0.8-mm interval. Eighty to 90 mL of iopromide (iodine concentration, 300 mg/mL) was injected intravenously at 2.5 mL/s, and imaging was performed 25–30 s after injection.

### Clinical and CT image features analysis

Image features were analyzed by a junior radiologist and reviewed by a medium-senior radiologist with 15–30 years of experience. The radiologists were blinded to the clinical findings and histological results. Disagreements between the two radiologists were resolved via discussion. Images were viewed at the lung (window width, 1,600 HU, and level, -600 HU) and mediastinal (window width, 350 HU, and level, 40 HU) windows. The CT features recorded in the analysis were as follows: (1) nodule location (left and right lungs, upper, middle and lower lobes); (2) diameter (average of the maximal long-axis and maximal short-axis perpendicular to it); (3) nodule–lung interface (clear or blurred/halo); (4) enhancement degree (compared with the muscle in the same slice, the enhancement degree of the nodule was low if the enhancement was lower than that of the muscle, medium if it was equal to that of the muscle, and high if it was higher than that of the muscle); and (5) other characteristics (lobulation, spiculation, vacuole, pleural indentation, air bronchogram, and vascular convergence). Clinical data of patients including sex, age, smoking history, and interval time between the CT scans and surgery were collected and recorded.

### Segmentation and radiomics feature extraction

This study used the Deepwise Multimodal Research Platform version 2.0.1.4 (https://keyan.deepwise.com, Beijing Deepwise & League of PHD Technology Co., Ltd, Beijing, China) to perform the radiomics analysis, which included image annotation, feature extraction and selection, and model establishment. The software is an integrated machine learning platform for medical data analysis based on the mature python Pyradiomics (version 3.0.1) and Scikit-learn (version 0.22) packages. Thin-slice CT images were uploaded to the platform in the original Digital Imaging and Communications in Medicine format. A blinded radiologist (Liu) manually delineated the regions of interest (ROI) of the nodules on all transverse images slice by slice, while avoiding the inclusion of adjacent vessels, bronchi, and normal lung tissue. Radiomics features were extracted from the ROIs of the uploaded CT images and analysis was quantified based on the volume of interest. To assess the interobserver reproducibility of the segmentation, 30 patients were selected randomly and re-segmented by the same radiologist, following the same principles as those described above, 1 week later. Intraclass correlation coefficients (ICCs) of features were calculated, and features with values > 0.75 were included in subsequent analysis. Normalization and resampling were used during image pre-processing with fixed bin width of 25, and all images were resampled to [1,1,0] after the B-spline interpolation sampling technology conducted in this study. High-throughput radiomics features extracted in this study included: first-order features, shape features, and texture features including gray level co-occurrence matrix (GLCM), gray level size zone matrix (GLSZM), gray level run length matrix (GLRLM), gray level dependence matrix (GLDM), and neighboring gray difference matrix (NGTDM). All features were named based on the three-level naming method, and each level was concatenated with “_” [19]. The first level was the image preprocessing method and specified parameters, the second level referred to the feature type, and the third level represented the statistical description.

### Feature selection and model construction

The original dataset was randomly divided into training and validation cohorts, in a ratio of 7:3, and a model was developed using fivefold cross validation. To alleviate redundancy between radiomics features, less significant features were removed when the linear correlation coefficient between any two features was greater than 0.9. The feature selection method used in this study was the analysis of variance *F*-test. Classification machine learning model was constructed using logistic regression to differentiate malignant from benign SSPNs. The clinical and CT characteristics in benign and malignant cohorts were firstly analyzed using univariate analysis, and characteristics with *p* < 0.05 in univariate analysis were further included in multivariate logistic regression. In addition, we constructed a logistic regression model that combined the selected radiomics and CT features. Radiomic signature was constructed through linear combinations of selected features by their respective coefficients, and the radiomics score was calculated as follows [20]:

Radscore = 1/(1 + exp(-logit))

$$\mathrm{logit }= {\beta }_{1} \times {\chi }_{1} + {\beta }_{2} \times { \chi }_{2} + {\beta }_{3} \times {\chi }_{3} + \dots + {\beta }_{n} \times {\chi }_{n}$$

where ${\beta }_{1}, {\beta }_{2}, {\beta }_{3}, \cdots , {\beta }_{n}$ are the coefficients of each feature and ${\chi }_{1}, {\chi }_{2}, {\chi }_{3}, \cdots , {\chi }_{n}$ are the magnitudes of the radiomics features.

Radscores was the decision probability of the logistic regression model, which indicated the relative risk of malignancy in the test samples. Finally, the performances of the radiomics, clinical, and combined models were compared statistically.

### Statistical analysis

Statistical analyses of clinical and CT features were performed using the SPSS software version 25.0 (IBM Corp., Armonk, NY, USA). Kolmogorov–Smirnov normality test was used to evaluate whether quantitative variables obey normal distribution. Continuous variables conforming to a normal distribution were expressed as the mean ± standard deviation; otherwise, they were expressed as the median (the first quantile; the third quantile). To compare the differences between groups, the Mann–Whitney *U* test and independent-samples t-test were used for quantitative variables, and Pearson’s chi-squared test was used for categorical variables. Model diagnostic performance was evaluated using the area under the receiver operating characteristic curve (AUC), accuracy, sensitivity, specificity, positive predictive value, and negative predictive value. The AUCs of different models were compared using the Delong test. All statistical tests were two-sided, and values of *p* < 0.05 were considered significant. A decision curve analysis (DCA) was used to calculate the clinical impact of the models.

## Results

### General information and CT characteristics

Detailed information and CT characteristics of the training and testing cohorts are shown in Table 1. A total of 324 patients with SSPNs (166 benign and 158 malignant) were selected. Among the 166 benign nodules, 125 were confirmed by postoperative histopathological results (including 16 granulomatous inflammations, 27 hamartomas, 36 pulmonary lymph nodes, 7 sclerosing pneumocytomas, and 39 incidences of nonspecific inflammation), and the remaining 41 nodules were proved to be benign at follow-ups. The median interval time between the CT scans and surgery was 16 (first quantile 9; third quantile 25) days. There were, in total, 158 malignant nodules, which included 22 minimally invasive adenocarcinomas, 125 invasive adenocarcinomas, 4 squamous cell carcinomas, 3 adenosquamous carcinomas, 3 carcinoids, and 1 small cell lung carcinoma. In the training cohort, there were significant differences among the benign and malignant groups in diameter, nodule-lung interface, lobulation, spiculation, vacuole, pleural indentation, air bronchogram, and vascular convergence.

**Table 1:** Demographic information and CT characteristics of training and testing cohorts

Characteristics	Training cohort (*n* = 226)			Testing cohort (*n* = 98)		
	**Benign** **(*n* = 116)**	**Malignant (*n* = 110)**	***p***	**Benign** **(*n* = 50)**	**Malignant (*n* = 48)**	***p***
Gender			0.319			0.658
Female	76 (65.5%)	65 (59.1%)		28 (56.0%)	29 (60.4%)	
Male	40 (34.5%)	45 (40.9%)		22 (44.0%)	19 (39.6%)	
Age (years)	56.4 ± 10.7	55.6 ± 9.5	0.572	56.7 ± 9.1	55.8 ± 9.8	0.629
Smoking history			0.981			0.307
Yes	22 (19.0%)	21 (19.1%)		12 (24.0%)	16 (33.3%)	
No	94 (81.0%)	89 (80.9%)		38 (76.0%)	32 (66.7%)	
Diameter (mm)	6.7 ± 1.9	8.4 ± 1.5	< 0.001	6.5 ± 1.8	7.9 ± 1.7	< 0.001
Location			0.281			0.857
RUL	23 (19.8%)	22 (20.0%)		7 (14.0%)	7 (14.6%)	
RML	19 (16.4%)	9 (8.2%)		10 (20.0%)	7 (14.6%)	
RLL	26 (22.4%)	24 (21.8%)		16 (32.0%)	13 (27.1%)	
LUL	17 (14.7%)	25 (22.7%)		8 (16.0%)	11 (22.9%)	
LLL	31 (26.7%)	30 (27.3%)		9 (18.0%)	10 (20.8%)	
Enhancement degree			0.477			0.864
Low	91 (78.4%)	93 (84.5%)		43 (86.0%)	41 (85.4%)	
Medium	13 (11.2%)	8 (7.3%)		3 (6.0%)	4 (8.3%)	
High	12 (10.3%)	9 (8.2%)		4 (8.0%)	3 (6.2%)	
Nodule-lung interface			< 0.001			0.003
Clear	111 (95.7%)	84 (76.4%)		48 (96.0%)	36 (75.0%)	
Blurred/Halo	5 (4.3%)	26 (23.6%)		2 (4.0%)	12 (25.0%)	
Lobulation	72 (62.1%)	93 (84.5%)	< 0.001	22 (44.0%)	41 (85.4%)	< 0.001
Spiculation	6 (5.2%)	49 (44.5%)	< 0.001	4 (8.0%)	17 (35.4%)	0.001
Vacuole	2 (1.7%)	22 (20.0%)	< 0.001	1 (2.0%)	4 (8.3%)	0.334
Pleural indentation 17 (14.7%)		54 (49.1%)	< 0.001	6 (12.0%)	20 (41.7%)	0.001
Air bronchogram 1 (0.9%)		23 (20.9%)	< 0.001	1 (2.0%)	11 (22.9%)	0.002
Vascular convergence 0 (0.0%)		6 (5.5%)	0.033	0 (0.0%)	5 (10.4%)	0.060

Data are expressed as number (%), or mean ± standard deviation

*LUL* Left upper lobe, *LLL* Left lower lobe, *RUL* Right upper lobe, *RML* Right middle lobe, *RLL* Right lower lobe

### Feature selection and model construction

There were significant differences between the benign and malignant groups in diameter, nodule-lung interface, spiculation, vacuole, pleural indentation, air bronchogram in the multivariate analysis (Table 2), and these six CT features were included to establish clinical logistic regression model. The AUC values of the clinical model in predicting risk of SSPNs were 0.920 (95% confidence interval [CI] 0.885−0.956) and 0.835 (95% CI 0.758−0.912) in the training and testing groups, respectively (Table 3).

**Table 2:** Multivariate analysis to identify significant factors for SSPNs

Features	*p*	Odds ratio	Lower	Upper
Diameter	< 0.001	1.450	1.192	1.764
Nodule-lung interface	< 0.001	10.996	3.852	31.388
Spiculation	0.001	4.079	1.730	9.615
Vacuole	0.001	9.752	2.396	39.696
Pleural indentation	< 0.001	3.782	1.888	7.576
Air bronchogram	0.001	13.820	2.929	65.213

**Table 3:** Diagnostic performance of the three prediction models in training and testing set

Group	Model	AUC (95% CI)	Accuracy	Sensitivity	Specificity	PPV	NPV
Training set	Clinical model	0.920 (0.885–0.956)	0.850	0.800	0.897	0.880	0.825
	Radiomics model	0.907 (0.875–0.938)	0.824	0.861	0.789	0.795	0.856
	Combined model	0.942 (0.918–0.966)	0.870	0.880	0.861	0.858	0.883
Testing set	Clinical model	0.835 (0.758–0.912)	0.745	0.604	0.880	0.829	0.698
	Radiomics model	0.900 (0.867–0.932)	0.815	0.848	0.783	0.788	0.844
	Combined model	0.930 (0.902–0.957)	0.858	0.867	0.849	0.846	0.870

*AUC* Area under the receiver operating characteristic curve, *CI* Confidence interval, *NPV* Negative predictive value, *PPV* Positive predictive value

**Fig. 2:**
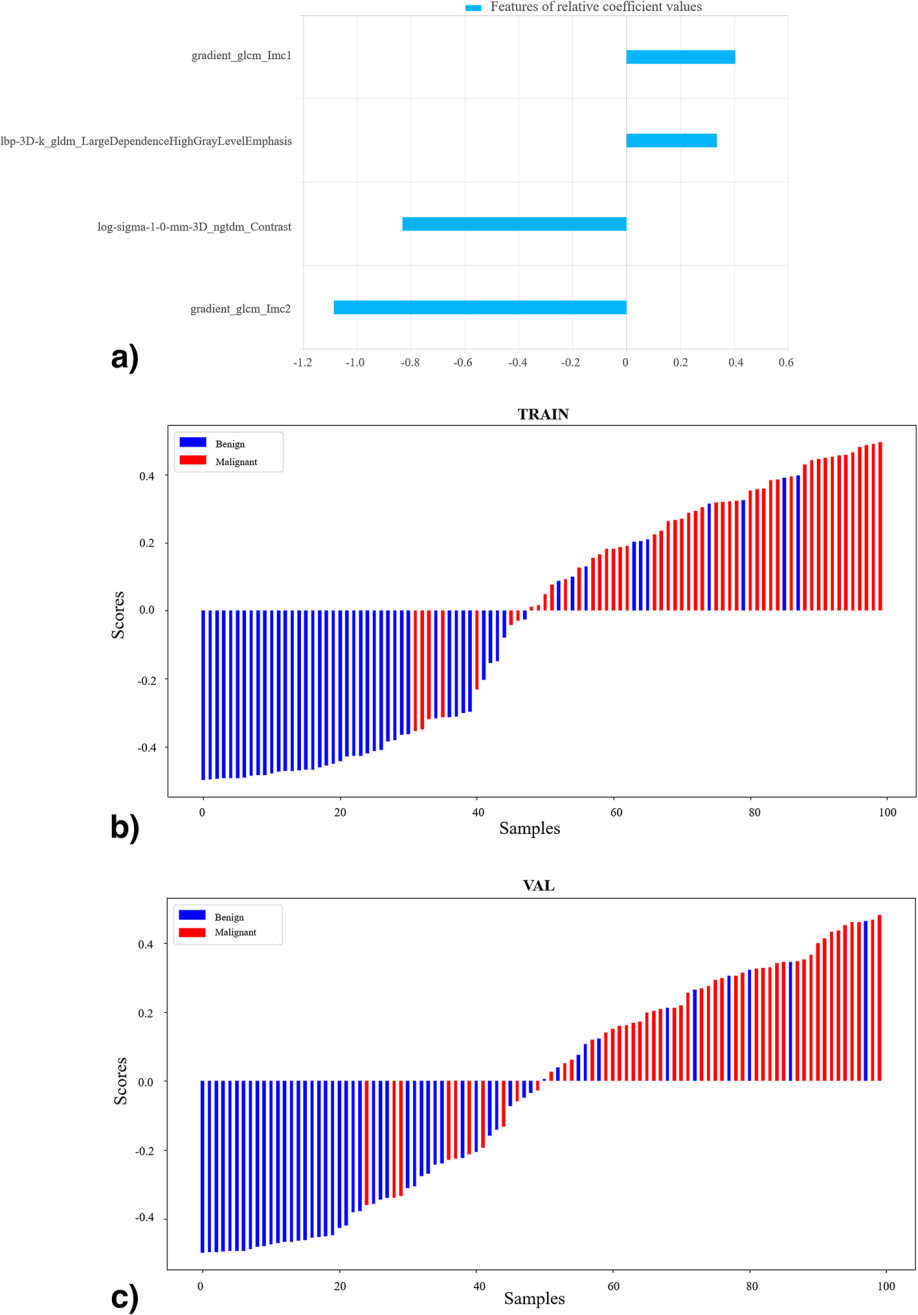
Feature coefficients and radiomics scores of the radiomics model. **a** The feature parameters and their corresponding coefficients of the radiomics model. The radiomics score graphs of the radiomics model in training group (**b**) and testing group (**c**). In **b** and **c**, the blue bars below baseline 0 represent benign nodules with correct prediction, the red bars above baseline 0 represent malignant nodules with a correct prediction, and the cross parts represent model prediction errors

**Fig. 3:**
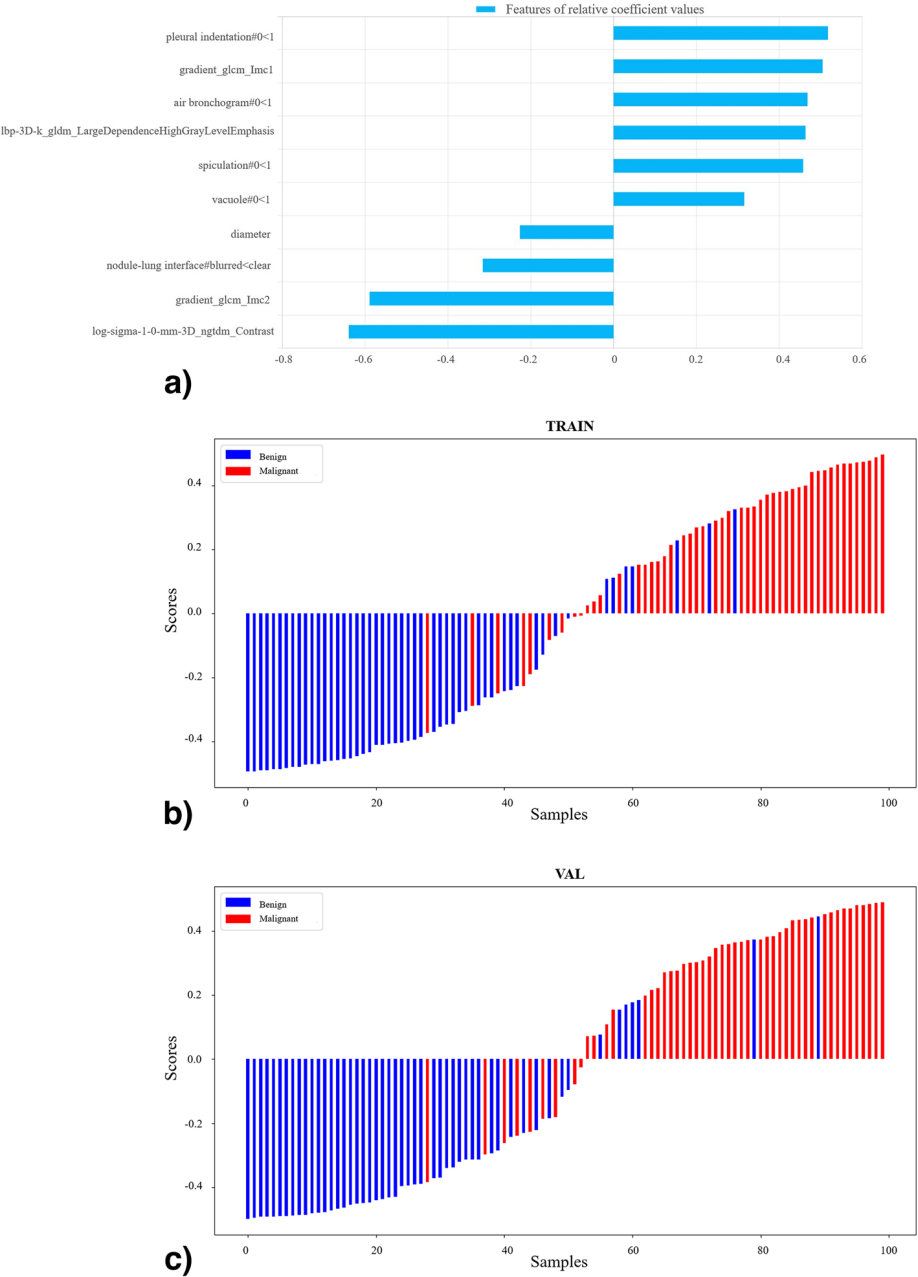
Feature coefficients and radiomics scores of the combined model. **a** The feature parameters and their corresponding coefficients of the combined model. The radiomics score graphs of the combined model in training group (**b**) and testing group (**c**)

A total of 2107 high-throughput radiomics features were extracted, and the four features that had the greatest relative weights were finally selected by *F*-test including gradient_glcm_Imc1, lbp-3D-k_gldm_LargeDependenceHighGrayLevelEmphasis, log-sigma-1–0-mm-3D_ngtdm_Contrast, and gradient_glcm_Imc2. The specific calculation formula for each feature can be found in the Pyradiomics web page (https://pyradiomics.readthedocs.io/). Figure 2 shows the radiomic features sorted by the absolute value of coefficients and rad-score distribution for benign and malignant nodules in the training and testing cohorts. The combined model was constructed by incorporating these six CT features and four radiomics features using logistic regression. Figure 3 shows the feature coefficients and the radiomics scores of the combined model in the training and testing cohorts.

### Model performance comparisons

**Fig. 4:**
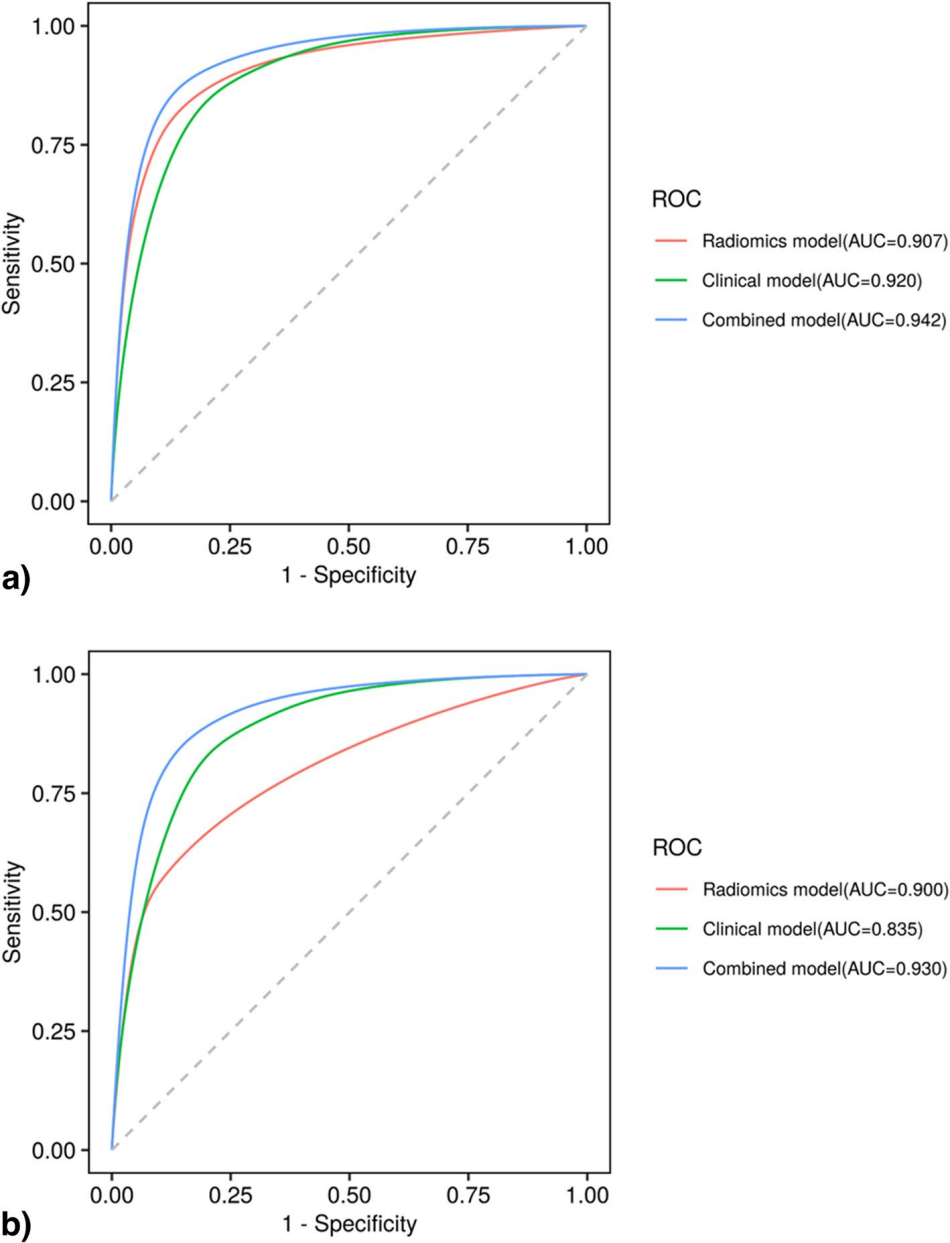
The ROC curves of the three prediction models for the classification benign and malignant SSPNs in training group (**a**) and testing group (**b**). *ROC*, Receiver operating characteristic; *SSPNs*, Subcentimeter solid pulmonary nodules

**Fig. 5:**
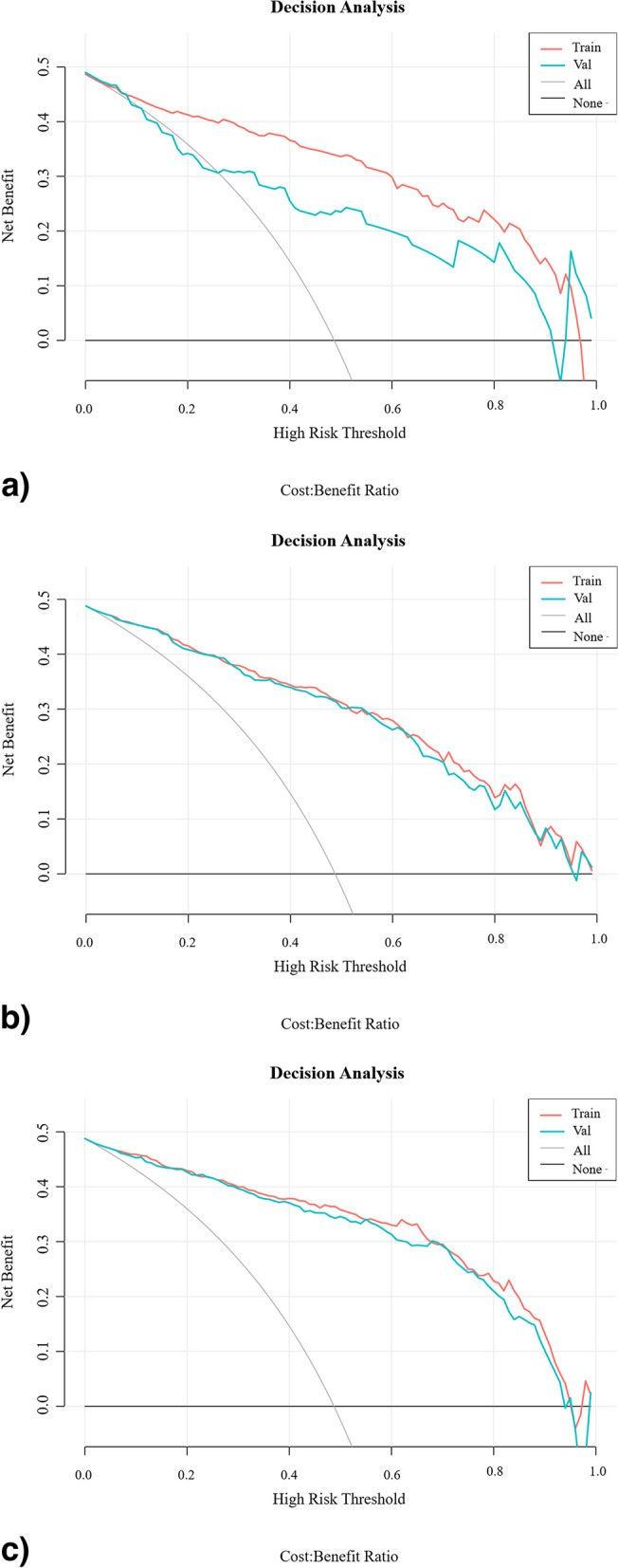
The decision curve analysis for clinical model (**a**), radiomics model (**b**), and combined model (**c**) in the training and testing group. The *x*-axis of the curves indicates the threshold probability. The *y*-axis indicates the net benefit. “All” and “None” show the hypothesis that all nodules were diagnosed as malignant or benign, respectively

**Fig. 6:**
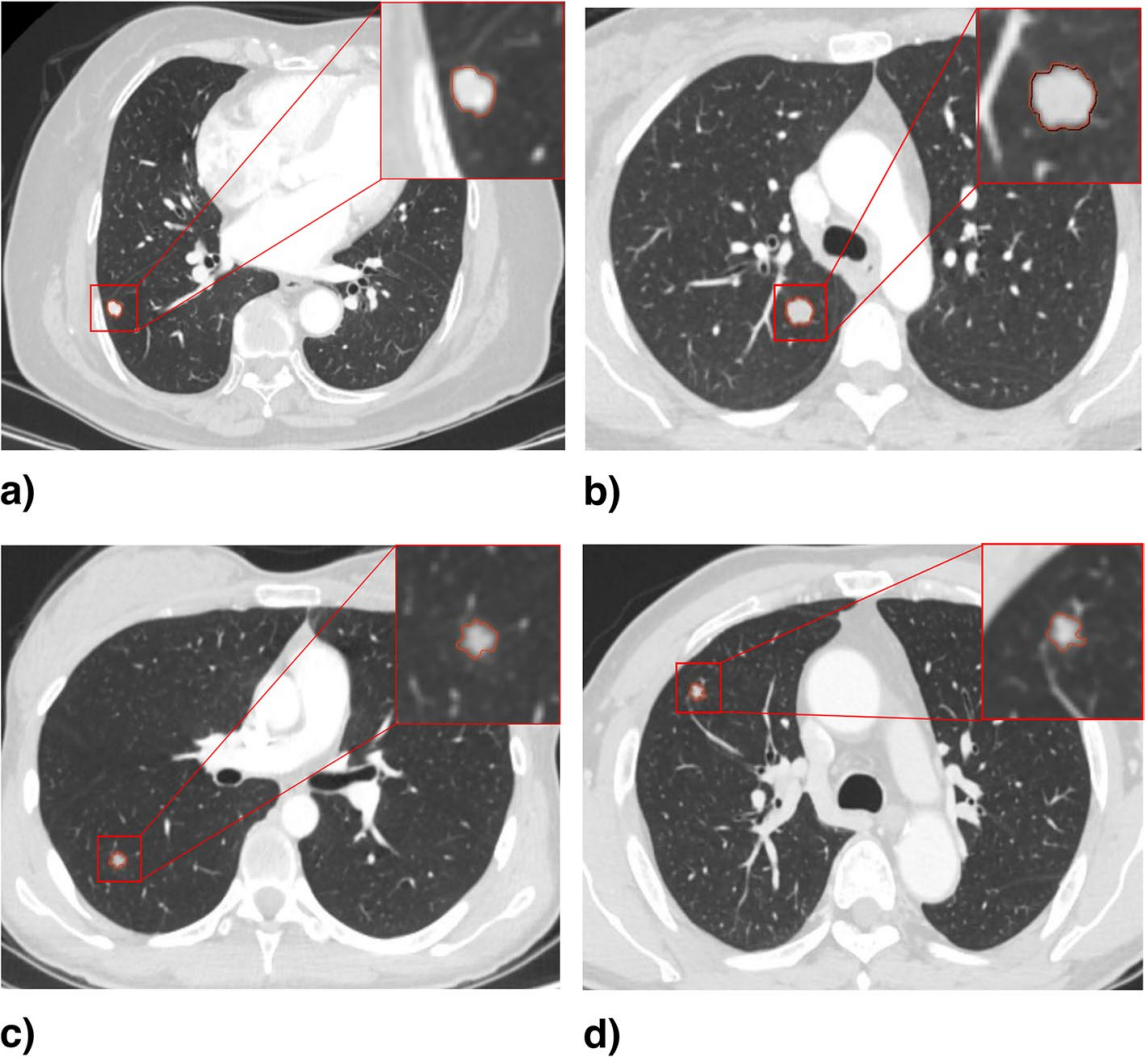
Representative segmentation results of nodules. **a** A nodule in RLL of a 75-year-old male, with mean diameter of 8.0 mm. The clinical model diagnosed it as malignant, while the radiomics and combined model diagnosed it as benign. Histopathological finding: sclerosing pneumocytoma. **b** A nodule in RUL of a 39-year-old male, with mean diameter of 10.0 mm. The clinical model diagnosed it as malignant, while the radiomics and combined model diagnosed it as benign. Histopathological finding: hamartoma. **c** A nodule in RUL of a 24-year-old female, with mean diameter of 6.0 mm. The clinical model diagnosed it as benign, while the radiomics and combined model diagnosed it as malignant. Histopathological finding: invasive adenocarcinoma. **d** A nodule in RUL of a 62-year-old male, with mean diameter of 6.5 mm. The clinical model diagnosed it as benign, while the radiomics and combined model diagnosed it as malignant. Histopathological finding: minimally invasive adenocarcinoma. *RLL*, Right lower lobe; *RUL*, Right upper lobe

Table 3 shows the diagnostic performance of the clinical, radiomics, and combined models in the training and test groups. ROC curves were drawn to compare the diagnostic accuracy of the clinical, radiomics, and combined models, as shown in Fig. 4. The combined model yielded the best predictive performance in the training (AUC 0.942; 95% CI 0.918−0.966) and test (AUC 0.930; 95% CI 0.902−0.957) groups. Based on the Delong test, the AUC of the combined model was significantly higher than the radiomics model in the training group (*p* < 0.001) and clinical model in the testing group (*p* = 0.025). In addition, the clinical model had lower sensitivity while the radiomics model showed lower specificity in the two groups. The combined model achieved both high sensitivity in training (0.880) and testing (0.867) groups and high specificity in training (0.861) and testing (0.849) groups. The rad-score distribution showed that compared with the radiomics model, the combined model had a relatively better overall prediction effect, as the combined model effectively increased the correct prediction of malignant nodules and reduced the risk of malignant nodules being misdiagnosed as benign, whether in the training or testing group. The DCA curves were drawn to investigate the clinical usefulness of the three models (Fig. 5). The representative SSPN segmentation results are shown in Fig. 6.

## Discussion

The differential diagnosis of benign and malignant solid pulmonary nodules has always been a difficulty in clinical work. For large solid nodules, findings such as over 10 mm or 15 mm in size, enhanced CT, PET-CT, CT functional imaging, puncture biopsy, and other methods can also be used to assist in diagnosis. However, most of these approaches are not effective for patients with SSPNs, for whom there is no better choice than planned follow-up. Unlike the indolent pathobiological behavior of subsolid nodules, solid nodules grow faster and are more likely to be invasive adenocarcinoma [21]. Mohammed et al. [22] proposed that among the patients with non-small cell lung cancer, 13%, 31%, and 46% had progression after 4, 8, and 16 weeks, and 3%, 13%, and 13% suffered from evident metastasis at 4, 8, and 16 weeks, respectively. Therefore, the delayed diagnosis of malignant solid nodules caused by follow-up may affect the prognosis of patients with early-stage lung cancer.

To assist radiologists in the early diagnosis of solid nodules non-invasively, the present study developed a machine learning model that was combined with radiomic features and CT imaging characteristics to differentiate malignant from benign sub-centimeter solid pulmonary nodules and verified its performance using ROC curves, AUC, accuracy, sensitivity, specificity, positive predictive value, and negative predictive value. The results revealed that the combined model provided better differentiation efficiency than the radiomics model and clinical model.

Clinical and CT features is the important basis for radiologists to diagnose the malignant from benign SSPNs. This study found that the mean diameter, nodule-lung interface, spiculation, vacuole, pleural indentation, and air bronchogram were independent predictors of SSPNs, in agreement with the findings of previous studies [13, 16, 23, 24]. This study found that the differences between benign and malignant nodules were statistically significant at pleural indentation, and the coefficient of this feature was the highest among CT features in the combined model. Pleural indentation is caused by contraction of the pleura in response to intratumor fibrosis, and malignant nodules with pleural indentation tend to be more aggressive, providing an important differential diagnostic sign in this study. The blurred nodule–lung interface of malignant nodules is due to the continuous infiltration of malignant nodules into the periphery during the growth process, while benign nodules generally exhibit no peripheral infiltration with a clear and smooth nodule–lung interface. Blurred interface is more common in small nodules, which may be related to the relative sparsity of tumor cells around the nodules [5]. Spiculation is invasive growth of the cancer and exudation or proliferative interstitial reaction, which is related to the active growth of tumor cells and the obstruction of connective tissue. Thin and short spiculation were more likely to be observed in malignant SSPNs than in benign SSPNs in this study. Although vacuole and air bronchogram were less frequently observed due to the small nodule size, the two signs were also conducive to the diagnosis of benign and malignant SSPNs. Smoking history was not an independent factor to discriminate malignant and benign SSPNs in this study, which may be because adenocarcinoma accounts for most malignant nodules and has a higher incidence in women. Compared to men, women rarely smoke in China [25]. The difference in CT enhancement between benign and malignant nodules was not obvious in this study, as there was an overlap of benign and malignant SSPNs, and benign nodules can also present increased enhancement due to blood flow, perfusion, or capillary permeability [26].

Radiomics can extract quantitative features from medical images with high throughput and reflect the internal heterogeneity of tumor tissues that cannot be observed by human eyes through objective and quantitative methods [16]. Several studies have found that radiomics models perform well in classifying malignant from benign solid pulmonary nodules; however, these studies only focused on specific pathological types, *i.e.*, lung adenocarcinoma and tuberculoma with nodule diameters less than 3 or 4 cm [11, 13, 15, 16]. Zhang et al. [16] proposed a diagnostic model combining CT and radiomic features and achieved an AUC of 0.85 (95% CI, 0.78–0.91) in the validation cohort, but they focused on solid nodules ranging from 5 to 20 mm. Therefore, there are still insufficient studies on radiomics models specifically targeting subcentimeter solid nodules containing different pathological types. Lin et al. [27] developed a radiomics model based on noncontrast enhanced CT images to distinguish benign and malignant SSPNs, with an AUC of 0.940 in the training cohort and 0.903 in the test cohort, but the study only included 180 nodules. This study expanded the sample size to 324, including a more comprehensive range of pathological types, such as adenocarcinoma, squamous cell carcinoma, adenosquamous carcinoma, carcinoid, small cell lung carcinoma, inflammation, hamartoma, pulmonary lymph node, sclerosing pneumocytoma, and other types.

The radiomics model developed for SSPNs in this study contained four features (gradient_glcm_Imc1, lbp-3D-k_gldm_LargeDependenceHighGrayLevelEmphasis, log-sigma-1–0-mm-3D_ngtdm_Contrast, and gradient_glcm_Imc2) and showed good diagnostic performance in differentiating malignant and benign SSPNs. GLCM describes texture by analyzing the spatial correlation characteristics of two pixels on an image that maintain a certain distance with each having a certain gray level. GLDM statistics describe the situation where the difference in texture between a certain pixel on an image and its surrounding pixels is less than a certain threshold. NGTDM quantifies the difference between a gray value and the average gray value of its neighbors within distance. The nodules in this study were segmented into three-dimensional volume of interest to extract the radiomic features, which can comprehensively reflect the internal information of the entire nodule. The efficacy of the radiomics model was comparable to the clinical model in the training group but superior to that of the clinical model in the testing group. The performance degradation of the clinical model may be due to uneven distribution of samples in the training and testing groups, but this finding also revealed that the diagnostic performance of the radiomics model was more stable than the clinical model. However, the specificity of the radiomics model was the lowest among the three models, and clinical model showed lowest sensitivity, especially in testing group (0.604). The combined model achieved the best discriminative ability among three models in training and testing groups with both high sensitivity and specificity, which make up for deficiencies of the above single models. The DCA also showed that the combined model has the best clinical application value, compared with the other two models.

As the technology of CT scanners has advanced, the spatial resolution of CT images has reached a higher level. Better spatial resolution can reveal tinier lung abnormalities, retrieve detailed information, and allow clear descriptions of lung anatomy, pathological changes, and disease states [28]. Albers et al. [29] proposed technical refinements of propagation-based imaging and achieved better image quality at lower x-ray dose levels, possibly revealing lung pathological lesions in 3D at high resolution. High spatial resolution makes it possible to visualize smaller structures and more details, which has a direct impact on accurate depiction of CT features and textural features in CT images. Studies have found that higher spatial resolution allows better differentiation of radiomics features and yields higher estimation accuracy for radiomics features [30, 31]. The increase in resolution may have the potential to improve the performance of our models based on CT and radiomics features, pending further experimental verification. This study has some limitations. First, this was a single-center retrospective study of nodules selected from a tumor hospital, which may lead to selection bias. Second, the model developed in this study was only based on enhanced CT images, and whether its efficacy is better than that of the plain CT-based radiomics model still needs to be further investigated in a comparative study. Third, there was a lack of external verification cohorts. It is necessary to conduct a multicenter study to validate the generalization ability of the models developed in this study. In addition, this study only focused on intratumoral radiomics features, and more image-derived information can be applied to model construction, such as the perinodular zone of the nodules and delta radiomics features [32, 33]. The performance of the model is expected to be further improved if these features can be incorporated.

In summary, radiomics signatures contribute to differentiating malignant from benign SSPNs, and the machine learning model that was constructed combining CT imaging characteristics and radiomics features in this study showed excellent diagnostic performance, with high sensitivity and specificity. The combined model that integrated the benefits of the clinical and radiomics models offers the potential to assist radiologists in diagnosing benign and malignant SSPNs.

## Data Availability

The datasets generated and analyzed during the current study are not publicly available due to data sharing not obtaining the informed consent of the patients but are available from the corresponding author on reasonable request.

## Abbreviations

AUC: Area under the receiver operating characteristic curve
CI: Confidence interval
CT: Computed tomography
SSPNs: Subcentimeter solid pulmonary nodules
